# Suffocating cancer: hypoxia-associated epimutations as targets for cancer therapy

**DOI:** 10.1186/1868-7083-3-9

**Published:** 2011-12-05

**Authors:** C Thirlwell, LKE Schulz, HK Dibra, S Beck

**Affiliations:** 1 Medical Genomics Laboratory, UCL Cancer Institute, 72, Huntley Street, WC1E 6BT, London

**Keywords:** Hypoxia, Hypoxia inducible factor (HIF), DNA methylation, histone modification, micro-RNA

## Abstract

Lower than normal levels of oxygen (hypoxia) is a hallmark of all solid tumours rendering them frequently resistant to both radiotherapy and chemotherapy regimes. Furthermore, tumour hypoxia and activation of the hypoxia inducible factor (HIF) transcriptional pathway is associated with poorer prognosis. Driven by both genetic and epigenetic changes, cancer cells do not only survive but thrive in hypoxic conditions. Detailed knowledge of these changes and their functional consequences is of great clinical utility and is already helping to determine phenotypic plasticity, histological tumour grading and overall prognosis and survival stratification in several cancer types. As epigenetic changes - contrary to genetic changes - are potentially reversible, they may prove to be potent therapeutic targets to add to the cancer physicians' armorarium in the future.

Here, we review the therapeutic potential of epigenetic modifications (including DNA methylation, histone modifications and miRNAs) occurring in hypoxia with particular reference to cancer and tumourigenesis.

## Introduction

### Tumour hypoxia - a regulatory factor in tumour growth

Control of cellular oxygen concentration is under strict regulation as hyperoxia causes damage secondary to reactive oxygen species and hypoxia leads to activation of a diverse array of downstream transcriptional pathways including angiogenesis, glucose metabolism and apoptosis.

It is well understood that tumourigenesis is dependent on the development of microvasculature for micronutrient supply and oxygenation. As this vasculature develops in a chaotic way with structural malformations, regions of hypoxia are present within all solid tumours. Normal oxygen tension in healthy tissue is 7% (53 mmHg), levels of oxygenation in tumours may vary from physiological levels (7%) to severe hypoxia (< 1%) which is usually found in areas adjacent to necrotic tissue [1]. It has also been reported that within the same region of a given tumour, levels of oxygenation may cycle due to poor vasculature and limited oxygen diffusion, resulting in intermittent periods of hypoxia [1]. These cyclical episodes of hypoxia lead to increased metastatic potential of cancer cells [2].

Cellular hypoxia is toxic, and when severe leads to cell death in both normal and cancerous cells. However, cancer cells have developed mechanisms through (epi)genetic modifications which allow them to survive and in some cases thrive in hypoxic conditions [3]. Preclinical studies have demonstrated that the selective pressure of tissue hypoxia drives the selective outgrowth of more aggressive tumour sub-clones [4]. In early stage lung cancer and pancreatic endocrine cancer markers of hypoxia have outperformed traditional histopathological staging for predicting prognosis [5,6].

The direct observation of tumour hypoxia through oxygen electrode recording was first made over ten years ago [7]. This study demonstrated the relationship between low oxygen tension and increased risk of metastasis along with poor prognosis in head and neck, breast and cervical cancers. It has since been possible to demonstrate hypoxia in vivo through the injection of pimidiazole [8].

**Figure 1:**
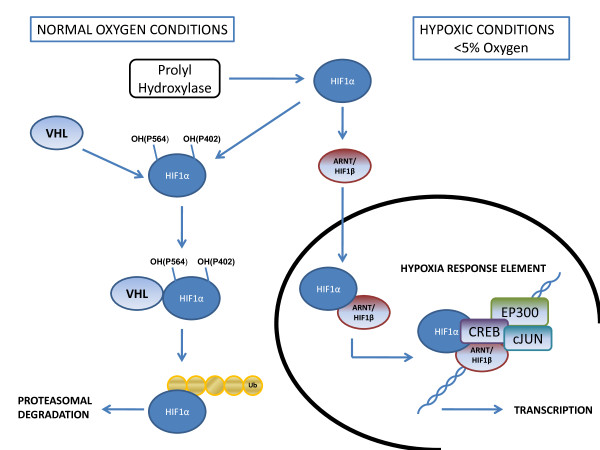
**Overview of the hypoxia inducible factor (HIF) pathway**. In normoxic conditions, HIF-1α binds to VHL at specific proline residues leading to ubiquitination and proteosomal degradation. During hypoxia, HIF-1α no longer binds to VHL and accumulates. This leads to formation of the hypoxia response element and subsequent transcription (HIF-1β also known as ARNT (aryl hydrocarbon receptor nuclear translocator), cJUN - jun proto-oncogene, CREB - cAMP responsive element binding protein 1, EP300 - E1A binding protein p300).

The hypoxia inducible factor (HIF) pathway is the key regulatory pathway activated in response to tissue hypoxia. In normoxic conditions HIF is degraded through interaction with the Von-Hippel Lindau (VHL) tumour suppressor protein (pVHL) where it becomes polyubiquinated and undergoes proteosomal degradation. The interaction between VHL and HIF is controlled via post-translational prolyl hydroxylation of HIF through prolyl hydroxylases (PHDs). In hypoxic conditions HIF does not bind to VHL due to the action of PHDs and therefore accumulates. HIF-1α then binds to hypoxia response elements (HRE) along with co-factors HIF-1β (also known as arylhydrocarbon receptor nuclear factor (ARNT)), E1A binding protein p300 (EP300), jun proto-oncogene (c-JUN) and cAMP responsive element binding protein (CREB). This leads to the transcription and up-regulation of over 100 genes involved in angiogenesis, glucose metabolism and transportation, erythropoietin production, cellular proliferation, tumour invasion/metastasis and *p53 *mediated apoptosis [9,10]. Three HIF-α subunits have been identified, HIF-1α and HIF-2α are thought to have roles in acute and chronic hypoxia respectively whereas the role of HIF-3α is yet to be determined [11]. *In vitro *studies in several cell culture systems have shown that HIF is activated at approximately 5% O_2 _(40 mmHg) and activity increases with decreasing oxygenation down to 0.2-1.0% (1.6-0.8 mmHg) O_2 _nearing anoxia [12]. See Figure 1 for an overview of the HIF pathway (adapted from Biocarta, http://www.biocarta.com). As HIF activation leads to the development of tumour vascularisation, it plays a significant role in tumour progression and metastasis. Over-expression of HIF-1α and HIF-2α has been documented in several primary tumours and associated metastases, the degree of expression correlates with angiogenesis, resistance to treatment and overall patient outcome [9,3]. The HIF pathway can also be activated through germline or somatic mutation of *VHL *or through *VHL *promoter methylation leading to transcriptional repression. Renal cell carcinoma can develop through all of these mechanisms (reviewed in [13,14]). For a review of the role of HIF in tumourigenesis see [12,3,15]. As HIF activation can be deleterious to both cancerous and normal cells, feedback mechanisms exist to control the level of HIF activation, which leads to a switch from HIF-1α to HIF-2α driven response when acute hypoxia becomes chronic. HIF-2α driven responses occurring in chronic hypoxia may be involved in the regulation of tumour cellular differentiation and stem cell maintenance (reviewed in [16]).

Alongside HIF related transcriptional response to tissue hypoxia, there is increasing evidence to support the development of hypoxia mediated epigenetic modifications which lead to further transcriptional changes and chromosomal instability. This is mediated through changes in DNA methylation at promoter regions and satellite repeats [17], histone modifications [18], and micro-RNAs [19]. Further genetic instability in hypoxia is due to gene amplification and DNA strand breaks at fragile sites alongside disruption of DNA repair [20].

Phenotypic transition can also occur as a result of hypoxia, with evidence of epithelial - mesenchymal transition (EMT) [21] and the development of tumour specific cancer stem cells (CSC), as seen in glioblastoma [1] and breast cancer [22]. CSCs are phenotypically similar to normal stem cells and behave similarly in that they are able to up-regulate DNA repair enzymes [23].

### Epigenetic manifestations of hypoxia in normal tissue

It is clear that the physiological response to hypoxia in normal tissues and organs must involve epigenetic modifications which lead to downstream changes in gene expression which have either protective or adaptive functions. The following studies have found evidence of a global increase in DNA methylation following a period of hypoxia with associated changes in transcriptional activation and repressive histone marks.

RNA expression array studies on murine placenta identified increased expression of DNA methyltransferase 3B (Dnmt3b), and methyl-CpG binding domain protein 1 (Mbd1) following a 48 hour period of hypoxia [24]. An increase in the expression of genes involved in metabolism, oxygen transport, proteolysis, cell death and reactive oxygen species metabolism was also identified. It was concluded from this study that hypoxia may contribute to long-term epigenetic changes in hypoxically stressed tissues and organs. Previous studies have demonstrated that reactive oxygen species can affect DNA methylation [25] suggesting a mechanism for the increased expression of DNA methyltransferases in hypoxia.

Increased expression of DNA methyltransferases during hypoxia in human tissues was confirmed when Watson *et al *determined the epigenetic signature of chronic hypoxia in normal prostate cell lines [26]. Following a 7 week period of normoxic culture, cells were then cultured for a further 4 weeks at 3% O_2 _and then 3 weeks at 1% O_2 _prior to assessment of global DNA methylation, DNMT and histone deacetylase activity. This found a global increase in DNA methylation with concomitant increase in DNMT3B expression and H3K9 histone acetylation. Gene-specific changes in DNA methylation were also observed at several key loci of imprinted genes.

Hypoxia is known to induce alterations in chromatin, including global deacetylation and also changes in histone methylation and acetylation in the promoter regions of hypoxia related genes. Chromatin precipitation analysis has identified a signature of chromatin modifications which is induced

under hypoxic stress. It has been suggested that this may play a role in gene regulation in proliferating tumour cells undergoing cyclic periods of hypoxia [27].

The jumonji-domain containing dioxygenases (JMJD) group of histone demethylases (JHDMs) have been recently described and are found to be regulated in hypoxic conditions through HIF-1α [28]. Within the JMJD group, JMJD1A and JMJD2B were found to be significantly up-regulated during hypoxia with JMJD2C showing modest up-regulation. This hypoxia related dynamic control of histone methylation is thought to regulate chromatin assembly and gene expression. JMJDs affect gene expression through demethylation of lysine residues of histone tails. In particular, JMJD1A has been found to demethylate H3K9me1 and H3K9me2 and JMJD2B demethylates H3K9me3. Transcriptional pathways have also been associated with individual JMJDs, JMJD1A is associated with the androgen receptor (AR) and regulates AR target genes. Both JMJD1A and JMJD2C regulate and increase the expression of genes involved in self-renewal and are targets for the transcription factor Oct-4 in embryonic stem cells[28].

Although all of these studies have documented a global increase in DNA methylation and histone modifications, which are associated with changes in downstream transcription, there are no studies to date which document the time course or order of these events. It is very likely that different cell and tissue types would require varying periods of hypoxia before "normal" hypoxic cells become "pre-cancerous" or "cancerous" hypoxic cells.

### Epigenetic manifestations of hypoxia in tumour development

#### DNA methylation

The observation of global hypomethylation was first observed in colorectal cancer cell lines in the 1980's [29]. Over the following twenty years a number of tumour suppressor genes were found to be silenced through promoter region hypermethylation [30]. The first DNA methylome (at single nucleotide resolution) was published in 2008, this was followed in quick succession by several other studies which have given us a wealth of information regarding the function of DNA methylation in both coding and non-coding regions of the genome [31]. For example, the concept of CpG island shores was developed (regions of lower CpG density occurring within 2 kb of CpG islands) [32]. These regions have since been found to harbour the majority of tissue specific differentially methylated regions of DNA [33].

There are few studies which have investigated genome-wide DNA methylation changes occuring during hypoxia. Methylation within DNA repeat elements (retrotransposable *Alu *and other short interspersed nuclear elements (SINEs)) is known to contribute to genomic stability, it has been postulated that chronic hypoxia can lead to global hypomethylation and subsequent increase in genomic instability and aneuploidy which is often observed in tumourigenesis. This is supported by two studies [17,34] reporting an increase in global hypomethylation in glioblastoma, sarcoma, colorectal cancer and melanoma cell lines when cultured in hypoxic conditions when compared to normoxic culture. The study of glioblastoma and sarcoma cell lines used RT-PCR methodology alongside bisulphite sequencing of *Alu *and other SINE repeat regions within the genome, whereas the study of colorectal cancer and melanoma cell lines used HPLC to quantify 5-methylcytosine (5-mC). These findings are the exact opposite of those observed when culturing non-cancerous cells in hypoxic conditions (ie an increase in global methylation) and may in part be due to a breakdown in the feedback mechanisms which exist to control the degree of HIF activation occurring in cancer cells and not normal cells during hypoxia.

#### Histone modifications

The most common histone modifications occurring in cancer are acetylation, deacetylation or methylation at lysine residues. These modifications are catalysed by histone deacetylases (HDACs) and histone methyltranferases (HMTs) [35]. Histone acetylation is most commonly associated with repression of transcription, whereas histone methylation is associated with both transcriptional activation and repression. A number of methylation reactions are associated with transcriptional activity (di- and trimethylation of H3K4 (H3K4me2 and H3K4me3), trimethylation of H3K36 (H3K36me3) and dimethylation of H3K79 (H3K79me2). In contrast, di- and trimethylation at H3K9, H3K27 and H4K20 is associated with gene silencing [36,37]. Specifically in cancer, a global loss of H4K16ac is seen alongside loss of the transcriptionally active mark H3K4me3 and gain of the transcriptionally repressive marks H3K9me and H3K27me [38].

##### Histone modifying enzymes play a key role in hypoxia-related tumourigenesis

It is increasingly evident that histone modifying enzymes play a key role in hypoxia-related tumourigenesis. Several research groups have demonstrated that hypoxia regulates the activity of histone demethylases [18]. Xia *et al *used ChIP-chip and RNA expression profiling of HepG2 (hepatocellular cancer cell line) and U87 (glioma cell line) cells grown in normoxic (ambient) and hypoxic (0.5% O_2 _for 4 hours) conditions to define HIF-1 chromatin binding targets. In total, 377 HIF-1 binding sites activated by hypoxia were identified across the genome. As predicted, many of the genes associated with these binding sites were from biological pathways known to be activated and controlled by HIF. However, enrichment for the 2-oxoglutarate dioxygenase family of enzymes was also observed including the previously described JHDMs. It was further demonstrated that up-regulation of these JHDMs helps to maintain histone homeostasis during hypoxic tumorigenesis [18]. The JHDM family of histone demethylases do not only require oxygen to function but are in some cases induced during hypoxia through HIF related mechanisms [11]. The JHDMs, JMJD2B and JMJD2C have been found to have oncogene-like propertied and are amplified in squamous cell of the oesophagus and medulloblastoma [39,40].

In addition, inactivating mutations in enzymes controlling histone modification have been detected in clear cell renal carcinoma (ccRCC), clear cell ovarian cancer [41] and pancreatic neuroendocrine tumours [42]. The histone modifying enzymes directly involved in ccRCC development are SET domain containing 2 *(SETD2)*, a histone H3 lysine 36 methyltransferase, Jumonji/ARID domain-containing protein 1C *(JARID1C)*, a histone H3 lysine 4 demethylase and ubiquitously transcribed X chromosome tetratricopeptide repeat protein *(UTX) *a histone H3 lysine 27 demethylase [43,44]. Even though a small fraction of clear cell renal cell carcinoma (3%) cases harboured inactivating mutations in *SETD2 *or *JARID1C*, these mutations are potentially of high relevance in hypoxia since 88% of samples with *SETD2 *and *JARID1C *inactivating mutations also harboured mutations in *VHL *and/or over-expression of egl nine homolog 3 *(ELGN3) *which is also known as HIF-prolyl hydroxylase 3 *(PHD3) *[43]. *JARID1B*, a histone H3 lysine 4 demethylase is found to be up-regulated in prostate cancer [45].

In gastric cancer, a hypoxia-induced epigenetic silencing of the tumour suppressor *RUNX3 *through histone H3-lysine 9 dimethylation and decreased H3 acetylation during disease progression has been observed [46]. *RUNX3 *is also known to be silenced in several cancers through promoter methylation. This study performed on gastric cancer cell lines revealed that following treatment with the histone deacetylase inhibitor trichostatin A and the cytosine-methylation inhibitor 5-aza-2-deoxycytidine, down-regulation of *RUNX3 *was reversed. Bisulphite-specific PCR demonstrated that promoter region methylation was unchanged in hypoxia, whereas chromatin immunoprecipitation (CHIP) found an increase in the repressive histone mark H3K9me2 and a decrease in the transcriptionally active H3K9 acetylation mark. It was also found that the G9a histone methyltransferase and HDAC1 were up-regulated following hypoxia, alongside diminished nuclear localisation and expression of *RUNX3*, demonstrating an epigenetic mechanism for the observed decrease in *RUNX3 *expression.

Hypoxic induction of histone demethylases therefore play a pivotal role in tumourigenesis, one such mechanism being HIF- α mediated induction of JHDMs leading to cancer progression. The role of histone methyltransferases which may oppose the effects of histone demethylases during hypoxia is less well understood. If the effects of these two opposing families of histone modifying enzymes during hypoxia are determined, a vital connection between environmental stressors and epigentically regulated tumourigenesis would be made and furthermore, novel epigenetic cancer therapeutic targets identified.

#### Micro-RNAs

Micro-RNAs (miRNAs) are short (20-24) non-coding nucleotide RNA molecules [47]. They are thought to regulate gene expression through control of mRNA turnover, inhibition of translation, promoter region activation and epigenetic silencing [48]. The "micro-transcriptome" is thought to account for 1-2% of the human genome and regulate the majority of translated genes [49]. miRNAs have the ability to "respond" both immediately and reversibly to hypoxic stress. miRNAs identified and implicated in tumorigenesis when normal tissue and tumour samples are compared [50] are affected through several factors in the tumour microenvironment such as hypoxia, pH, glucose metabolism and paracrine growth factors. Hypoxia regulated miRNAs are implicated in both renal cell cancer an glioblastoma development, several other hypoxia regulated miRNAs are in development and cancer bioamarkers, these are discussed below.

##### Hypoxia related mi-RNAs can be used as diagnostic and prognostic cancer biomarkers

Kulshreshtha *et al *have identified a group of hypoxia regulated miRNAs (HRMs). Several of these miRNAs regulate apoptotic signalling during hypoxic periods. The majority of the identified HRMs were found to be over-expressed in human cancers and were consistently induced in breast and colorectal cancer cell lines during culture in hypoxic conditions [51,52]. Other groups have also reported induction of miRNAs as a response to hypoxia [53] but it is difficult to compare studies directly due to variation in culture times and degree of hypoxia used for culturing between research groups.

miR-210 is known to be regulated by hypoxia and has found to be a diagnostic marker in pancreatic adenocarcinoma [54] and prognostic marker in breast cancer [55]. miR-210 is also over-expressed in late stage non-small cell lung cancer [56]. Subsequent *in vitro *studies on the lung adenocarcinoma cell line A549 demonstrated that miR-210 expression results in caspase 3/7 activity, followed by mitochondrial dysfunction. In the same study, subunit D of succinate dehydrogenase complex (SDHD) was validated as a miR-210 target [56].

##### The relationship between the HIF pathway and hypoxia-mediated miRNAs

Neal *et al *first demonstrated the impact of the *VHL *tumour suppressor gene on miRNAs in renal clear cell carcinoma [57]. This study elegantly showed that miRNAs are downstream effectors of the HIF-induced hypoxia response. Under hypoxic conditions (or through *VHL *mutation or *VHL *promoter methylation), miR-210 expression is significantly increased and potentially targets the iron-sulphur cluster protein (ISCU). ISCU is implicated in the mitochondrial electron transport chain and potentially, in anaerobic respiration of tumours. A further group of miRNAs has been identified which regulate *VEGF *(miR-16, 20a, 20b, 17-5p, 27a, 106a, 106b, 107, 193a, 210, 320, 321) [58].

The over expression of miR-21 (a HRM) has been implicated in the development of glioblastoma (which is known to have extensive regions of hypoxia and necrosis), and knock-down of this miRNA in glioblastoma cell lines lead to apoptosis, suggesting a role in tumour survival [59].

Although none of the hypoxia regulated miRNAs described above are used routinely for clinical diagnosis and prognosis to date, these biomarkers once validated, are easily accessible from both tissue biopsies, resections and are found in circulating plasma enabling clinical access for both diagnostic and prognostic purposes.

### Potential epigenetic targets for hypoxia related cancer therapy

#### DNA methyltransferase inhibitors

DNA methyltransferase (DMNT) inhibitors have been investigated for some years as a novel class of anti-cancer drug. There is substantial evidence to support the use of the DNMT inhibitors 5-azacytidine and 5-aza-2'-deoxycytidine (decitabine) for certain haematological malignancies, resulting in their approval by the FDA. Following a number of phase I and II trials the evidence for their use in solid tumours is less encouraging. In light of this, these agents have not been trialled with specific reference to tumour hypoxia. Treatment with decitabine has been shown to lead to re-expression of *VHL *in clear cell renal carcinoma in vitro and in vivo through inhibition of DNA methylation in the *VHL *promoter region [60]. This leads to restoration of the normal physiological HIF pathway and reduction of accumulation of HIF-1α and downstream hypoxia response element related transcription. Re-expression of BNIP3 which is associated with hypoxia mediated apoptosis following treatment with decitabine has also been observed in pancreatic adenocarcioma cell lines [61].

#### Histone Deacetylase Inhibitors

HDAC inhibitors are compounds which function through inhibition of deacetylases, they are a diverse group of compounds which are divided in to classes I-IV. HDAC inhibitors do not act solely as inhibitors of HDACs as a large number of non-histone transcription factors and transcriptional co-regulators are known to be modified by acetylation.

A clear mechanism of action of these drugs is yet to be elucidated. To date, they have been found to have pro-apoptopic and anti-angiogenic properties. Several HDAC inhibitors are currently under evaluation in clinical trials both a single agents and in combination with chemotherapry and targeted therapy [62-64]. The second generation HDAC inhibitor Vorinostat has been approved by the FDA for the treatment of cutaneous T cell lymphoma.

Recently it has been shown that the class I and II HDAC inhibitors disrupt HIF both directly through reduction of HIF-1α levels and indirectly through repressing transcription, the exact mechanism of this remains unknown [65]. A number of HDAC inhibitors have been reported to down-regulate HIF-1α stability in cancer cell lines. It has been suggested that the transcription complexes of *HIF-1α *and *HIF-2α *require modification by type I/II deacetylases in order to become transcriptionally active, it is through this mechanism that HDAC inhibitors are thought to reduce HIF transcription [66]. Fath *et al *further demonstrated that the inhibition of *HIF-1α *and *HIF-2α *transactivation mediated by HDAC inhibitors was independent of VHL and p53 function. This is a significant finding as it implies that HDAC inhibitors may have a therapeutic effect in VHL deficient tumours (such a renal cell cancer and pancreatic neuroendocrine tumours). The class III HDAC inhibitor SirtI has also been found to deacetylate *HIF-1α *and *HIF-2α *thus repressing HIF activity [67].

Sodium butyrate, a novel HDAC inhibitor has been shown to inhibit hypoxia induced *HIF1α *induction and inhibited in vitro and in vivo angiogenesis [68]. The study demonstrated that sodium buyrate treatment of endothelial cells down-regulated both HIF1α and VEGF protein levels thereby regulating angiogenesis. This indicates that HDACs are involved in oxygen dependant gene expression and angiogenesis.

##### Combination of HDAC inhibitors with other targeted treatments can have an additive effect

Verheul *et al*., demonstrated in prostate and renal cancer cell lines that combination treatment with the mTOR inhibitor rapamycin and the HDAC inhibitor LBH589, significantly reduced HIF-1α protein expression and consequently suppressed tumour induced angiogenesis on comparison with either agent used singularly [69].

The effects of HDAC inhibitors on both HIF degradation and transcription are therapeutically very appealing and further clinical evaluation should be considered trialing other targeted treatments such as mTOR and VEGF inhibitors alongside HDAC inhibition. It should be noted however that long term systemic administration of HDAC inhibitors may lead to downstream effects on erythropoeisis and ischaemia (including coronary ischaemia) through inhibition of the physiological role that HIF plays in erythoropoietin production, and adaptive metabolic changes that occur during hypoxia. Therefore scheduling and timing of these therapies requires robust phase I trial assessment.

#### mi-RNA targeted therapy

Through the identification of specific miRNAs involved in tumorigenesis (in both normoxic and hypoxic conditions) it is deemed feasible to inhibit specific miRNAs in the future through anti-miRNA oligonucleotides [70], locked nucleic acid-modified oligonucleotides [71] and antagomirs [72]. To date, none of these agents have progressed further than phase I clinical trials.

There is recent evidence supporting the potential use of anti-oxidants and naturally occurring compounds to regulate miRNA expression in cancer cells [73]. Li *et al *[74] demonstrated down-regulation of miR-135a and miRNA 135b in colorectal cancer cell lines (which target the 3' untranslated region of adenomatous polyposis coli gene) through treatment with mistletoe lectin-I, the mechanism of this is thought to be through degradation of the miRNA precursors. Anti-oxidants such as Se-Methylselenocysteine (MSC) and N-acetyl cysteine (NAC) have been shown to inhibit HIF-1α in solid tumours in mouse models [75,76], historically it was thought that anti-oxidants had an anti-tumourigenic effect through the reduction of reactive oxygen species (ROS) and subsequent DNA damage, these studies demonstrate a direct inhibition of HIF by anit-oxidants. MSC when administered alongside irinotecan chemotherapy improved clinical outcome in mice with human squamous cell tumour xenografts through inhibition of HIF-1α [75] and NAC was shown to reduce HIF activity in mouse models of hepatocellular carcinoma and B cell lymphoma [76].

The role of miR-210 in relation to tumour hypoxia has been recently reviewed [77]. Alongside its potential use as a novel hypoxia diagnostic marker in pancreatic cancer [54,78] and prognostic biomarker in breast [55], head and neck [79] and renal cancer [57]. Although these studies do not demonstrate a direct mi-RNA targeted therapy, they illustrate a novel potential of these epigenetic markers within a clinical setting to help aid cancer diagnosis via non-invasive methods and ultimately impact on patient treatment and prognosis. Table 1 gives an overview of tumourigenic hypoxia related epigenetic changes and potential therapeutic strategies and targets.

**Table 1:** Tumourigenic hypoxia related epigenetic changes and potential therapeutic strategies and targets.

	Modification observed in hypoxia related tumourigenesis	Therapeutic potential
**DNA methylation**	Global DNA hypomethylation	• DNMT inhibition in haematological malignancies • Re-expression of specific tumour suppressor genes (eg. VHL) through decitabine therapy

**Histone modification**	• Hypoxic regulation of histone demethylase activity • Up-regulation of HDAC1 and histone methyltransferase	• HDAC inhibitors disrupt HIF through altered transcription and degradation • Combination of HDAC inhibiton with mTOR inhibition has additive effect *in vitro*

**miRNAs**	mi-RNAs associated with diagnosis and prognosis eg mi-210	• Selected hypoxia related mi-RNA inhibition through - Anti-miRNA oligonucleotides - Antagomirs • Indirect regulation of miRNA through HIF inhibitors

## Conclusions and outlook

It is clear that epigenetic changes occurring during hypoxia have a significant downstream effect physiologically in normal tissue, in the transition from normal to cancerous tissue and in the progression of solid tumours. These changes can be used as biomarkers in diagnosis and prognosis, for example the micro-RNA miR-210 in pancreatic and breast cancer.

However, in order to fully understand these changes and harness their clinical benefit for diagnostics and therapeutics, several key areas need to be addressed which are discussed below.

In normal tissue global DNA hypermethylation is observed following a period of hypoxia whereas in cancer cell lines global DNA hypomethylation is observed leading to increased genomic instability. This is likely due to decreased DNA methyltransferase activity - both maintenance and *de novo*. What we do not know is whether the global DNA hypomethylation observed in hypoxic cancer tissue is somehow protective enabling survival in hypoxic conditions.

It is not known whether a stepwise chain of epigenetic events occurs during hypoxia, ie changes in DNA methylation occurring before histone modifications and alteration in expression of downstream mi-RNAs. If this sequence of events could be determined it might highlight the best diagnostic and therapeutic strategies to employ in different cancer types. In reality it is likely that there is interaction and positive and negative feedback between all epigenetic modifications.

Therapeutically, DNMT inhibitors have not been trialled with respect to tumour hypoxia. However HDAC inhibitors appear to have a direct effect on both *HIF *transcription and degradation making them promising therapeutic agents for use in vascular tumours and tumours with severe regions of hypoxia. If used in the right clinical setting for selected tumour types this therapeutic intervention could prove to be clinically beneficial. Future therapeutic strategies could include the integration of HDAC inhibition with other targeted therapies such mTOR and VEGF/c-kit/platelet derived growth factor (PDGF) tyrosine kinase inhibitors. Sequencing and duration of treatment with such therapies in combination is yet to be determined.

With the recent massive expansion of molecular biology tools available for studying the epigenome (both array and sequencing based) [80], we are at a turning point in furthering our understanding of the effects of hypoxia on the epigenome and identifying novel clinical biomarkers and therapeutic targets.

## Competing interests statement

The authors declare that they have no competing interests.

## Authors' contributions

CT drafted and revised the manuscript, LKES contributed to content relating to miRNAs and HKD contributed to content relating to histone modifications. SB provided guidance for the overall structure and content of the manuscript. All authours read and approved the final manuscript.
